# Pop in, pop out: a novel gene-targeting strategy for use with CRISPR-Cas9

**DOI:** 10.1186/s13059-015-0810-2

**Published:** 2015-11-10

**Authors:** Ralf Kühn, Van Trung Chu

**Affiliations:** Max Delbrück Center for Molecular Medicine, Robert Rössle Strasse, 13125 Berlin, Germany

## Abstract

The CRISPR-Cas9 system is frequently used to create small deletions in the genomes of mammalian cells, but the isolation of precisely targeted mutants is still challenging. A new, two-step ‘pop in & out’ targeting approach facilitates this task.

See related Research article: http://www.genomebiology.com/2015/16/1/231

## Gene editing in mammalian cells using CRISPR-Cas9

Genetic engineering in mammalian cells is flourishing in recent years thanks to the use of sequence-specific nucleases that create double-strand breaks (DSBs) in genes of interest, enforcing the repair of the disrupted sequences. Proof-of-principle was provided by methodologies involving zinc-finger nucleases and TALENs, both of which have been superseded by the more versatile CRISPR-Cas9 gene-editing system [1]. This system is composed of the nuclease Cas9, which is guided to specific DNA sequences by short complementary single guide RNAs (sgRNAs) to create targeted DSBs. Gene editing at DSBs is mediated by cellular DNA repair mechanisms, either the imprecise non-homologous end joining (NHEJ) pathway (mutNHEJ), which leads to small deletions, or homology-directed repair (HDR), which utilizes an homologous DNA molecule as a repair template, leading to precise nucleotide insertions or replacements. In cell lines such as HEK293, CRISPR-Cas9 can be used efficiently to generate knockout alleles that result from small, frame-shifting deletions; these deletions reach high frequencies upon transfection with Cas9 and sgRNA expression vectors [2]. By contrast, HDR repair [3], being restricted to the S and G2 phases of the cell cycle, requires an additional co-transfected gene-targeting vector or single-stranded oligonucleotide, and as a result targeted knock-in alleles that have been modified by HDR are obtained at substantially lower frequencies. In specific experimental settings, the recovery of targeted clones may be further complicated by the resistance of cell lines to transfection procedures, inaccessibility of the target locus or a limited efficacy of individual sgRNAs. Thus, methods and protocols for the enrichment of targeted cells are increasingly demanded to avoid the expansion and screening of large numbers of clones. The recent work of Thomas Cech and colleagues published in *Genome Biology* [4] provides a new and smart solution that can accomplish this task.

## Enrichment and isolation of mutant cells

Three ways to enrich targeted cells within or from Cas9-transfected populations have been described to date. In the first approach, cells are transfected with an additional fluorescent reporter gene or a nuclease reporter construct, allowing the fluorescence-activated cell sorting (FACS) enrichment of successfully transfected cells, which are subsequently cloned and genotyped [5, 6]. Alternatively, the activity of NHEJ key molecules, such as DNA ligase IV, can be suppressed in transfected cultures, leading to a global increase of HDR events in the cell population [7, 8]. The timed delivery approach uses cultures synchronized for the S phase of the cell cycle for transfection, in order to maximize the proportion of cells that are able to undergo HDR repair [9]. Nevertheless, none of these methods allows the direct selection of cells that harbor the desired targeted allele, unless the HDR template vectors also include selection markers such as drug resistance or reporter genes. The use of drug-selectable gene-targeting vectors is well established and mandatory for gene targeting in mouse embryonic stem (ES) cells [10] because the frequency of spontaneously occurring HDR, without assistance by site-specific nucleases, is very low. The approach described by Cech and colleagues [4] uses the classic targeting-vector design in a new way to improve the isolation of targeted clones induced by CRISPR-Cas9 gene editing. Cech and colleagues were troubled by the low frequencies of CRISPR-Cas9-induced HDR in the telomerase reverse transcriptase gene (*TERT*) in HEK293 and other cell lines. To overcome this problem, Cech and colleagues designed a two-step ‘pop in & out’ targeting strategy using vectors that contained a fluorescent marker gene and FACS for the isolation of targeted clones, followed by the removal of the selection marker in the second step.

## A simple ‘pop in & out’ targeting approach for use with CRISPR-Cas9

**Fig. 1:**
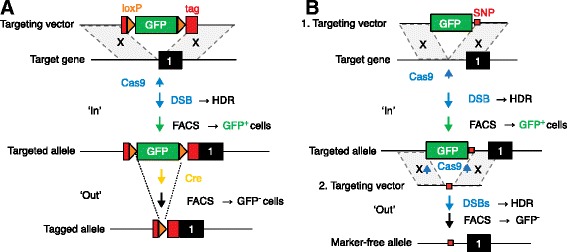
Two step, ‘pop in & out’ approach for the generation of CRISPR-Cas9-induced targeted mutants. **a** In the ‘in’ step, the targeting vector (homology regions shaded *gray*) introduces a tag segment that is disrupted by a loxP-flanked green fluorescent protein (*GFP*) reporter gene. A Cas9 and sgRNA-induced double strand break (*DSB*) stimulates homology-directed repair (*HDR*) and enables the enrichment of targeted GFP^+^ cells by fluorescence-activated cell sorting (*FACS*). In the ‘out’ step, the marker is deleted by Cre/lox-mediated recombination and GFP^−^ cells are subsequently enriched by FACS. **b** Two step in & out targeting approach for the seamless removal of a marker gene. In the first step, the targeting vector introduces a nucleotide replacement (a single nucleotide polymorphism (*SNP*)) next to a GFP reporter. The marker is removed from the targeted allele using Cas9 and a pair of sgRNAs that recognize the end of the marker cassette. The marker gene is removed by HDR with a targeting vector to provide sequences (homology regions shaded) that are wild type except for the SNP, and GFP^−^ cells are subsequently enriched by FACS

In the first application of the pop in & out approach (Fig. 1a), Cech and colleagues set out to add an N-terminal tag to the TERT protein to enable its visualization. The targeting vector included TERT homology regions and the tag-coding segment, interrupted with a loxP-flanked green fluorescent protein (GFP) gene that allowed the isolation of cells harboring either random or recombined stable genomic vector integrations. Random vector integrations usually occur in only approximately 1 in 10,000 transfected cells; consequently, this background could be outcompeted by the stimulatory effect on HDR of co-transfected Cas9 and a *TERT*-specific sgRNA, which occurs in a larger fraction of cells. Indeed, Cech and colleagues found that 84 % of the clones established from the FACS-enriched GFP^+^ population contained targeted *TERT* alleles. In the second step of the in & out procedure, the loxP-flanked GFP marker could be excised from the targeted locus by transient expression of Cre recombinase and the enrichment by FACS of GFP-negative cells (Fig. 1a). All of the clones established from the GFP^−^ fraction had lost the marker gene, resulting in tagged alleles that could be used to study *TERT* localization. A single loxP site of 34 bp remained within the targeted allele, which could be tolerated as an additional segment within the tag-coding region. In studies in which the insertion of a loxP sequence within a coding region needs to be avoided, vector design could be easily modified by placing the GFP marker into a neighboring intron region.

In the second application of the pop in & out approach (Fig. 1b), Cech and colleagues aimed to introduce a single base-pair replacement into the *TERT* promoter region, in order to test for the functionality of a single nucleotide polymorphism (SNP), without leaving any unrelated sequence trace in the genome. The vector for the first, ‘in’ targeting step included a GFP marker gene next to the desired nucleotide replacement. Targeted ‘in’ clones were established from the GFP^+^ cell population isolated by FACS. In the second, ‘out’ step, the marker could be completely removed upon transfection with Cas9 and a pair of sgRNAs that cut at both ends of the GFP gene, followed by HDR with a marker-free targeting vector containing only the SNP mutation (Fig. 1b). GFP-free clones were isolated by Cech and colleagues from GFP^−^ cells enriched by FACS, but a background of silenced, GFP^−^ cells made the identification of targeted clones less efficient. Nevertheless, the ‘out’ step, which enables the seamless excision of the GFP gene from the targeted locus while preserving the SNP mutation, is a new, smart alternative to classic marker removal by Cre recombinase leaving a loxP site in the genome.

The work of Cech and colleagues capitalizes on the flexibility of targeting vector design, which is well established in the field of mouse ES cells, and combines it with CRISPR-Cas9 gene editing. While most applications of CRISPR-Cas9 aim to introduce desired mutations in a single targeting step, low recombination efficiencies may necessitate major efforts to reach this goal. In these cases, the two-step in & out procedure is a more rational way to obtain targeted mutants, although it requires a second cycle of vector construction and clone isolation. Thus, the in & out targeting approach adds to the versatility of CRISPR-Cas9-mediated genome engineering and offers a new method for the direct selection of cells harboring targeted mutations. In future, it may be further combined with the suppression of NHEJ repair or the timed delivery approach, simplifying targeted mutagenesis for the benefit of genetic research.

## Conclusions

Genome engineering using the CRISPR-Cas9 system is becoming increasingly popular and its applications are continuously expanding. The two-step gene-targeting strategy developed by Cech and colleagues is a new development that facilitates the generation of precisely modified knock-in alleles in mammalian cells.

## Abbreviations

DSB: Double-strand break
ES: Embryonic stem cell
FACS: Fluorescence-activated cell sorting
GFP: Green fluorescent protein
HDR: Homology-directed repair
NHEJ: Non-homologous end joining
sgRNA: Single guide RNA
SNP: Single nucleotide polymorphism
*TERT*: Telomerase reverse transcriptase gene
